# Behavior-aware deep reinforcement learning for multi-objective outpatient scheduling optimization

**DOI:** 10.1038/s41598-026-50893-5

**Published:** 2026-06-04

**Authors:** Xiaoyu Wan, Xiayan Zhang, Weiqun Weng, Pu Han, Xiangyun Xu

**Affiliations:** 1 Outpatient Department, Tongzhou Bay People’s Hospital, Nan’tong, 226333 Jiangsu China; 2 Nuesing Department, Tongzhou Bay People’s Hospital, Nan’tong, 226333 Jiangsu China

**Keywords:** Outpatient scheduling, deep reinforcement learning, behavioral science, prospect theory, multi-objective optimization, patient satisfaction, Engineering, Mathematics and computing, Psychology, Psychology

## Abstract

Outpatient departments in large hospitals face persistent scheduling inefficiencies characterized by prolonged patient waiting, underutilized resources, and high no-show rates. Existing scheduling approaches largely ignore the behavioral heterogeneity of patients, treating satisfaction as a simple proxy of waiting time rather than a psychologically grounded construct. This paper proposes MO-SAC-B, a multi-objective deep reinforcement learning framework that integrates behavioral science theory into the scheduling optimization process. We first construct a behavior-driven discrete-event simulation environment that encodes prospect-theoretic waiting disutility, nonlinear patience decay, and behaviorally calibrated no-show and abandonment dynamics. A satisfaction-aware reward shaping mechanism translates these behavioral constructs into dense learning signals, while a multi-objective Soft Actor-Critic algorithm with adaptive weight adjustment and prioritized experience replay navigates the efficiency–satisfaction Pareto frontier. Experiments calibrated with real outpatient data from a tertiary hospital demonstrate that MO-SAC-B reduces mean waiting time by 21.9%, improves composite patient satisfaction by 12.7 points, and lowers the no-show rate by 25.8% relative to the strongest baseline. Ablation studies confirm that each behavioral component contributes meaningfully, with synergistic effects amplifying performance gains under high patient flow conditions. Robustness analysis further validates the framework’s adaptability to demand surges and resource disruptions.

## Introduction

Large-scale general hospitals in China face a persistent operational bottleneck: outpatient departments routinely serve thousands of patients each day, yet the scheduling infrastructure supporting these visits has evolved only incrementally over the past two decades. Extended waiting remains the single most reported source of dissatisfaction among outpatient visitors, with average wait-to-service ratios exceeding 3:1 in many tertiary institutions^1^. The mismatch between surging demand and constrained clinical capacity creates cascading delays across registration, consultation, examination, and pharmacy stages, degrading both patient experience and resource utilization^2^. This problem is not unique to China—hospitals worldwide grapple with analogous congestion—but the sheer volume of daily visits in Chinese public hospitals amplifies the urgency of finding systematic, data-driven scheduling solutions.

Early attempts to address outpatient scheduling drew heavily on classical queuing theory. Models grounded in M/M/c and M/G/1 frameworks offered closed-form insights into server utilization and expected waiting time under idealized arrival assumptions^3^. While analytically elegant, these formulations struggled to capture the stochastic complexity of real outpatient environments, where patient arrivals are non-stationary, service durations vary across specialties, and no-show behavior introduces unpredictable capacity slack. To relax some of these restrictive assumptions, researchers turned to metaheuristic and heuristic optimization techniques—genetic algorithms, simulated annealing, and tabu search—that could handle combinatorial scheduling problems with greater flexibility^4^. Such approaches improved upon static models by exploring larger solution spaces, yet they typically operated in an offline mode, producing schedules before the clinic day begins and offering limited capacity to adapt once real-time disruptions materialize.

The advent of data-driven predictive modeling and deep learning methods opened a new avenue for outpatient scheduling research, complementing the optimization and search-based techniques described above that also fall under the broader machine learning umbrella. Supervised learning models demonstrated the ability to predict appointment durations, no-show probabilities, and patient acuity levels with reasonable accuracy, thereby informing more realistic schedule construction^5^. Discrete-event simulation, often combined with optimization modules, became a popular methodology for evaluating scheduling policies under various what-if scenarios^6^. More recently, reinforcement learning (RL)—particularly deep reinforcement learning (DRL)—has attracted growing attention as a paradigm capable of learning adaptive, sequential decision policies directly from interaction with a simulated or real environment^7^. DRL methods such as Deep Q-Networks and Proximal Policy Optimization have been applied to operating room scheduling, emergency department triage, and appointment sequencing, showing promising gains in throughput and wait-time reduction^8^.

Despite this progress, several critical gaps persist. First, the vast majority of existing scheduling models treat patients as homogeneous entities characterized solely by arrival time and service requirement. In practice, patient behavior is markedly heterogeneous: individuals differ in punctuality, tolerance for waiting, propensity to abandon queues, and sensitivity to perceived fairness^9^. Behavioral science research has long documented phenomena such as the peak-end rule in retrospective satisfaction evaluation, reference-dependent preferences, and asymmetric reactions to gains versus losses in waiting contexts^10^. Yet these behavioral insights have seldom been formally integrated into scheduling optimization frameworks. Second, real-time dynamic rescheduling under non-stationary conditions remains technically challenging. Most RL-based studies rely on simplified state representations that omit crucial contextual variables—downstream congestion, time-of-day effects, and cross-department dependencies—limiting their transferability to complex multi-stage outpatient workflows. Third, patient satisfaction, though frequently cited as an objective, is rarely quantified in a manner amenable to direct optimization. Satisfaction proxies such as total waiting time or makespan fail to reflect the subjective, nonlinear, and context-dependent nature of patient experience^11^.

These shortcomings motivate our effort to bridge behavioral science theory and deep reinforcement learning within a unified scheduling framework. Behavioral science provides the theoretical lens for modeling how patients perceive, evaluate, and react to the waiting process, while DRL supplies the computational machinery for learning high-quality scheduling policies in high-dimensional, stochastic environments. Their integration is not merely additive; behavioral models reshape the reward signal that guides policy learning, enabling the agent to optimize for experiential outcomes rather than purely operational metrics. We argue that this coupling is both timely and necessary, given the increasing availability of electronic medical record data and real-time location tracking in modern hospitals, which supply the fine-grained observations on which such models depend^12^.

This paper makes three principal contributions that collectively distinguish our work from prior multi-objective reinforcement learning (MORL) studies. Our novelty does not reside in extending SAC to the multi-objective case per se—such extensions have appeared in the MORL literature^53,54^—but rather in the integration of behaviorally grounded reward signals, drawn from prospect theory and patience psychology, into a multi-objective DRL scheduling framework, an integration that has not been attempted in prior outpatient scheduling research. Contribution 1: we construct a behavior-driven patient flow simulation environment encoding heterogeneous patient types, stochastic service processes, and multi-stage routing, informed by established behavioral constructs including prospect-theoretic waiting disutility and the peak-end heuristic. Contribution 2: we design a satisfaction-aware reward shaping mechanism that translates behavioral satisfaction models into dense, informative reward signals, enabling the RL agent to optimize for experiential quality rather than purely operational proxies. Contribution 3: we propose a multi-objective SAC algorithm with adaptive weight adjustment and prioritized experience replay to navigate the efficiency–satisfaction Pareto frontier. We additionally compare MO-SAC-B against both conventional scheduling baselines and dedicated MORL algorithms (Envelope MOQ-Learning, Pareto DQN, and a PPO-based multi-objective variant) to demonstrate that the behavioral embedding, rather than the choice of RL backbone alone, drives the observed performance gains. Through extensive simulation experiments calibrated with real outpatient data, we demonstrate that the proposed framework yields substantial improvements in both operational efficiency and patient satisfaction.

## Theoretical foundations and problem modeling

### Behavioral science analysis framework for outpatient patient flow

Outpatient waiting is not merely a temporal phenomenon but a psychologically mediated experience. Prospect theory, originally proposed for decision-making under uncertainty, offers a compelling lens through which to model how patients evaluate waiting episodes^13^. A central tenet is that individuals assess outcomes relative to a reference point rather than in absolute terms, and they exhibit pronounced asymmetry between perceived losses and equivalent gains. In the outpatient context, patients typically arrive with an expected waiting duration $\:{w}_{ref}$, shaped by prior visits, appointment confirmations, or social norms. When the actual wait * w* exceeds this reference, the deviation is experienced as a loss weighted more heavily than a comparable time saving would be valued as a gain^14^.

We formalize this subjective waiting disutility through a reference-dependent value function adapted from prospect theory, expressed as a standard piecewise power formulation:

1 $$\:V\left(w\right)=\left\{\begin{array}{ccc}({w}_{ref}-w{)}^{\alpha\:},&\:w\le\:{w}_{ref}\text{}-\lambda\:(w-{w}_{ref}{)}^{\beta\:},&\:w>{w}_{ref}\end{array}\right.$$

where $\:\alpha\:,\beta\:\in\:\left(\mathrm{0,1}\right)$ govern the curvature of the value function in the gain and loss domains respectively, and $\:\lambda\:>1$ denotes the loss aversion coefficient capturing the amplified sensitivity to excess waiting^15^. Research on time perception psychology further suggests that waiting tolerance degrades nonlinearly as elapsed time grows. We therefore define a patience decay function:

2 $$\:\varPhi\:\left(t\right)={e}^{-\eta\:(t-{w}_{ref}{)}^{+}}$$

where $\:(t-{w}_{ref}{)}^{+}=\mathrm{m}\mathrm{a}\mathrm{x}(0,t-{w}_{ref})$ and $\:\eta\:>0$ controls the decay rate^16^. This exponential formulation reflects empirical observations that patient frustration escalates sharply once waiting surpasses a subjectively tolerable threshold.

Building on these constructs, the probability that a patient abandons the queue—either through no-show before arrival or balking mid-visit—can be modeled as a behaviorally grounded function. For no-show behavior, we specify:

3 $$\:{P}_{ns}\left(d\right)=\frac{1}{1+{e}^{-({\gamma\:}_{0}+{\gamma\:}_{1}d+{\gamma\:}_{2}X)}}$$

where * d* represents the appointment lead time, * X* encapsulates patient-specific covariates (e.g., visit history, distance), and $\:{\gamma\:}_{0},{\gamma\:}_{1},{\gamma\:}_{2}$ are estimable parameters^17^. For mid-visit abandonment, the instantaneous departure hazard at time * t* integrates the patience function with current queue state:

4 $$\:h\left(t\right)={h}_{0}\cdot[1-\varPhi\:(t\left)\right]\cdot(1+\delta\:\cdot Q(t\left)\right)$$

Here $\:{h}_{0}$ is a baseline hazard rate, $\:Q\left(t\right)$ denotes the observable queue length at time * t*, and $\:\delta\:$ captures the contagion effect whereby longer visible queues amplify departure inclination^18^. Equation (1) through (4) collectively furnish the behavioral substrate for the simulation environment developed in subsequent sections, grounding scheduling optimization in empirically defensible models of patient cognition rather than purely mechanical arrival–service abstractions.

### Mathematical modeling of the outpatient scheduling problem

We consider an outpatient network comprising * K* departments, each equipped with $\:{C}_{k}$ service windows (consultation rooms) staffed by physicians with heterogeneous service rates. Patients arrive according to a non-homogeneous Poisson process with time-varying intensity $\:\lambda\:\left(t\right)$, reflecting the well-documented surge patterns observed in morning clinic hours^19^. Upon arrival, each patient * i* carries a priority class $\:{p}_{i}\in\:\{\mathrm{1,2},\dots\:,P\}$ and a routing sequence determined by a department transition probability matrix $\:R=[{r}_{kl}{]}_{K\times\:K}$, where $\:{r}_{kl}$ denotes the probability of referral from department * k* to department * l*^20^. Service durations at department * k* follow a log-normal distribution $\:{S}_{k}\sim\mathrm{L}\mathrm{o}\mathrm{g}\mathrm{N}({\mu\:}_{k},{\sigma\:}_{k}^{2})$, a choice supported by empirical fitting studies on outpatient consultation times^21^.

The scheduling decision at each epoch assigns waiting patients to available windows. Let $\:{x}_{ijk}\left(t\right)\in\:\left\{\mathrm{0,1}\right\}$ indicate whether patient * i* is assigned to window * j* in department * k* at time * t*. The multi-objective optimization model seeks to simultaneously minimize average patient waiting time, maximize departmental resource usage, and maximize aggregate patient satisfaction. Formally, the three objectives are:

5 $$\:{f}_{1}=\mathrm{m}\mathrm{i}\mathrm{n}\frac{1}{N}\sum\:_{i=1}^{N}\sum\:_{k=1}^{K}{W}_{ik}$$

6 $$\:{f}_{2}=\mathrm{m}\mathrm{a}\mathrm{x}\frac{1}{K}\sum\:_{k=1}^{K}\frac{\sum\:_{i}^{}{S}_{ik}}{\sum\:_{j=1}^{{C}_{k}}{T}_{jk}}$$

7 $$\:{f}_{3}=\mathrm{m}\mathrm{a}\mathrm{x}\frac{1}{N}\sum\:_{i=1}^{N}{U}_{i}$$

where $\:{W}_{ik}$ is the waiting time patient * i* experiences at department * k*, $\:{S}_{ik}$ is the actual service duration, $\:{T}_{jk}$ represents the total available operating time of window * j* in department * k*, and $\:{U}_{i}$ denotes the composite satisfaction score for patient * i* computed through the behavioral value function in Eq. (1)^22^. These objectives inherently conflict—aggressive utilization may inflate waiting, while satisfaction depends nonlinearly on waiting as established in Sect.  2.1.

The model is subject to several operational constraints. Physician workload must remain bounded:

8 $$\:\sum\:_{i}^{}{x}_{ijk}\left(t\right)\cdot{S}_{ik}\le\:{L}_{jk}^{\mathrm{m}\mathrm{a}\mathrm{x}},\forall\:j,k$$

where $\:{L}_{jk}^{\mathrm{m}\mathrm{a}\mathrm{x}}$ is the maximum allowable cumulative service burden for physician * j* in department * k*. Concurrently, room capacity and priority precedence must hold:

9 $$\:\sum\:_{i}^{}{x}_{ijk}\left(t\right)\le\:1,\forall\:j,k,t;{W}_{ik}\le\:{W}_{{i}^{{\prime\:}}k}\mathrm{i}\mathrm{f}{p}_{i}<{p}_{{i}^{{\prime\:}}}$$

The first condition ensures single-patient occupancy per window at any instant; the second enforces that higher-priority patients receive earlier service within the same department queue^23^. This constrained multi-objective formulation, combined with the behavioral parameters from Sect.  2.1, defines the optimization landscape over which the deep reinforcement learning agent operates in subsequent sections.

### Fundamental framework of deep reinforcement learning

The outpatient scheduling problem defined in Sect.  2.2 naturally maps onto a Markov Decision Process (MDP), specified by the tuple $\:(\mathcal{S},\mathcal{A},\mathcal{P},\mathcal{R},\gamma\:)$, where $\:\mathcal{S}$ is the state space, $\:\mathcal{A}$ the action space, $\:\mathcal{P}$ the transition dynamics, $\:\mathcal{R}$ the reward function, and $\:\gamma\:\in\:\left[\mathrm{0,1}\right)$ the discount factor^24^. An agent interacts with this environment by selecting actions according to a policy $\:{\pi\:}_{\theta\:}\left(a\right|s)$ parameterized by $\:\theta\:$, aiming to maximize the expected cumulative return:

10 $$\:J\left(\theta\:\right)={\mathbb{E}}_{{\pi\:}_{\theta\:}}\left[\sum\:_{t=0}^{T}{\gamma\:}^{t}{r}_{t}\right]$$

Two paradigms dominate deep reinforcement learning. Value-based methods estimate state-action values and derive policies implicitly, while policy gradient methods optimize $\:J\left(\theta\:\right)$ directly through gradient ascent on the policy parameters^25^. Pure policy gradients, however, suffer from high variance. Proximal Policy Optimization (PPO) addresses this by constraining updates within a trust region via a clipped surrogate objective:

11 $$\:{L}^{PPO}\left(\theta\:\right)={\mathbb{E}}_{t}\left[\mathrm{m}\mathrm{i}\mathrm{n}\left({\rho\:}_{t}\left(\theta\:\right)\hat {{A}}_{t},\mathrm{c}\mathrm{l}\mathrm{i}\mathrm{p}\left({\rho\:}_{t}\right(\theta\:),1-\epsilon,1+\epsilon)\hat {{A}}_{t}\right)\right]$$

where $\:{\rho\:}_{t}\left(\theta\:\right)=\frac{{\pi\:}_{\theta\:}\left({a}_{t}\right|{s}_{t})}{{\pi\:}_{{\theta\:}_{old}}\left({a}_{t}\right|{s}_{t})}$ is the importance sampling ratio and $\:\hat {{A}}_{t}$ the generalized advantage estimate^26^. The clipping mechanism prevents destructively large policy shifts, a property particularly valuable in scheduling environments where abrupt policy changes can cascade through downstream queues.

Soft Actor-Critic (SAC) takes a different route by incorporating an entropy bonus into the objective, encouraging exploration while maintaining stable convergence. Its augmented objective reads:

12 $$\:{J}_{SAC}\left(\theta\:\right)={\mathbb{E}}_{{\pi\:}_{\theta\:}}\left[\sum\:_{t=0}^{T}{\gamma\:}^{t}\left({r}_{t}+{\alpha\:}_{H}\mathcal{H}\left({\pi\:}_{\theta\:}\right(\cdot\left|{s}_{t}\right))\right)\right]$$

where $\:\mathcal{H}(\cdot)$ denotes the entropy of the policy and $\:{\alpha\:}_{H}$ is a temperature coefficient that can be tuned automatically^27^. SAC’s entropy regularization proves especially advantageous for the multi-objective scheduling landscape formulated in Eqs. (5)–(7), where multiple near-optimal scheduling strategies may coexist and premature convergence to a single mode would be undesirable.

Both PPO and SAC share a critical strength for our problem setting: they handle continuous or large discrete action spaces, adapt to non-stationary dynamics through ongoing interaction, and require no explicit model of transition probabilities—an important practical consideration given the stochastic, behaviorally complex patient flow environment constructed in this study^28^. We adopt these two algorithms as candidate policy optimizers in Section III.

It is worth situating the present work within the broader MORL landscape, which has matured considerably in recent years. Hayes et al.^53^ provided a practical guide to MORL and planning, categorizing solution concepts by whether the utility function is known, unknown, or partially specified, and recommending algorithm families accordingly. Felten et al.^54^ introduced a decomposition-based taxonomy (MORL/D) that bridges multi-objective optimization and reinforcement learning, offering a unified vocabulary for classifying scalarization, Pareto, and indicator-based approaches. Preference-conditioned methods have also advanced rapidly: PD-MORL^55^ demonstrated that a single policy network, conditioned on a preference vector, can cover diverse regions of the Pareto front in continuous control tasks. Abels et al.^36^ proposed dynamic weight adjustment for multi-objective deep RL, a mechanism we adapt in our Eq. (18). Despite these algorithmic advances, applications of preference-aware MORL to healthcare scheduling remain scarce. Most existing healthcare RL studies rely on single-objective formulations or ad hoc scalar reward functions without systematic multi-objective decomposition^7,8^. Our work fills this gap by embedding behaviorally grounded preferences directly into a multi-objective SAC framework, allowing the scheduling agent to navigate the efficiency–satisfaction trade-off in a principled manner rather than through manual weight tuning.

## Design of the behavior-aware deep reinforcement learning scheduling model

### Construction of the behavior-driven outpatient patient flow simulation environment

The simulation environment is built on a discrete-event simulation (DES) engine, a paradigm well suited for modeling systems where state transitions occur at irregular, event-driven intervals rather than fixed time steps^29^. Four functional modules compose the core architecture. The patient arrival module generates individual patients according to the non-homogeneous Poisson process specified in Sect.  2.2, assigning each patient a priority class, behavioral profile (reference waiting time, loss aversion coefficient ), and a routing sequence sampled from the transition matrix. The triage and queuing module receives arriving patients, routes them into department-specific priority queues, and manages queue ordering based on the priority rules encoded in Eq. (9). The consultation service module simulates physician–patient interactions, drawing service durations from department-specific log-normal distributions and releasing patients upon completion. Finally, the department referral module determines whether a patient proceeds to another department or exits the system, governed by the stochastic routing probabilities. In this architecture, the state representation at each decision epoch aggregates real-time queue lengths, window occupancy indicators, per-department waiting time statistics, and patient-level behavioral features (reference waiting time and loss aversion coefficient) into a single vector fed to the RL agent. The action output specifies a patient-to-window assignment decision, accompanied by priority reordering and room activation adjustments. The reward signal combines an efficiency penalty for idle resources, a satisfaction component computed through the prospect-theoretic value function, and a constraint violation penalty, as detailed in Sect.  3.2.$\:{w}_{ref}\lambda\:R{r}_{kl}$

**Fig. 1:**
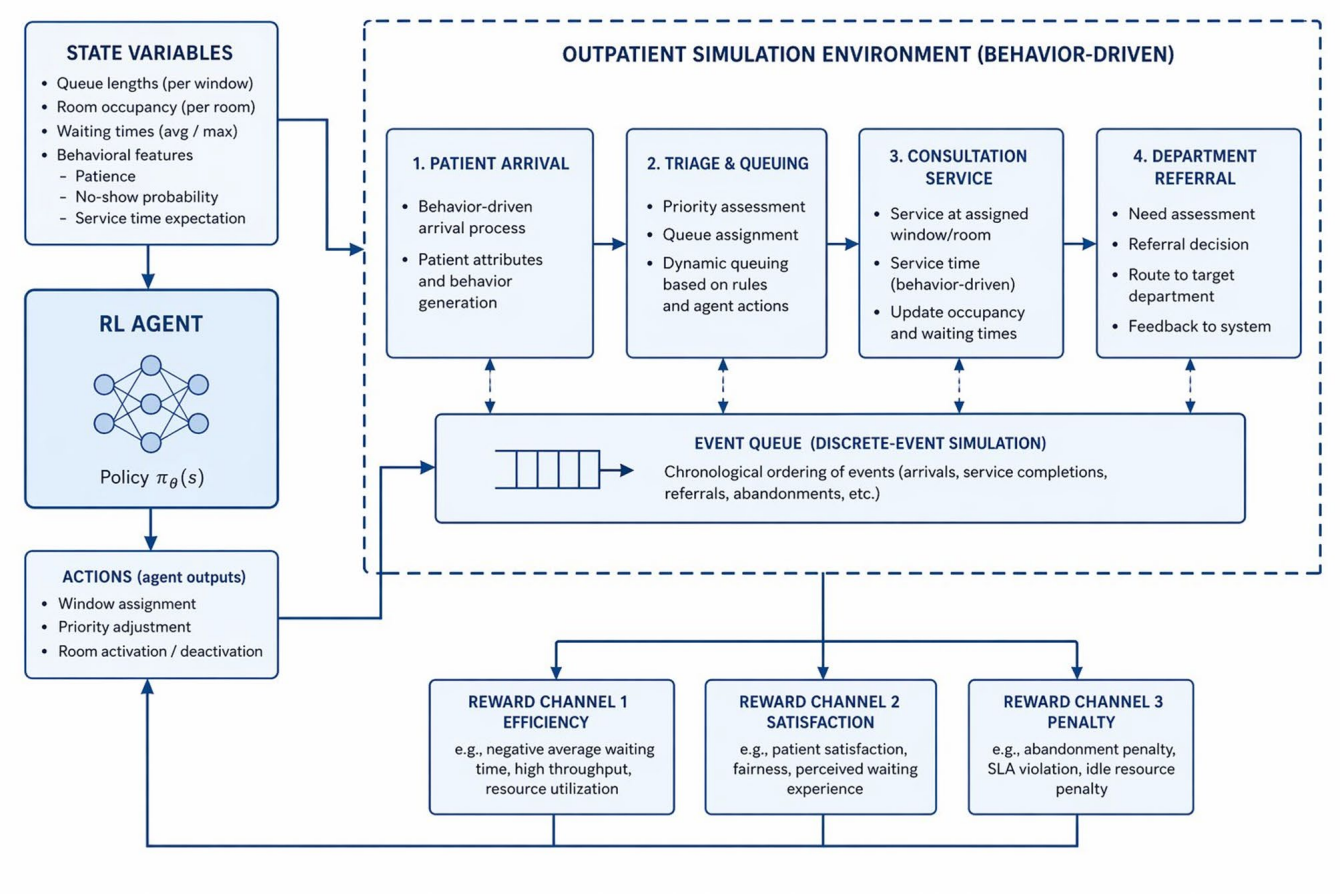
Architecture of the behavior-driven outpatient patient flow simulation environment.

What distinguishes this environment from conventional DES models is the explicit embedding of behavioral dynamics. At each simulation tick, the no-show model in Eq. (3) probabilistically removes scheduled patients who fail to appear, while the mid-visit abandonment hazard in Eq. (4) evaluates whether waiting patients depart prematurely^30^. Simultaneously, the satisfaction tracker updates each patient’s cumulative subjective disutility via Eq. (1), producing a real-time satisfaction trajectory that feeds directly into the reward computation. As Fig. 1 illustrates, the overall environment architecture connects these four modules through an event queue managed by a next-event time advance mechanism, where the simulation clock jumps to the earliest pending event—be it an arrival, service completion, referral, or behavioral departure^31^.

The interface between the simulation environment and the RL agent follows the standard Gymnasium API convention. At each decision epoch—triggered whenever a service window becomes idle—the environment exposes a state vector encoding current queue lengths, average waiting times per department, window availability, and aggregate satisfaction scores. The agent returns an action specifying which patient to assign to the freed window. The environment then advances until the next decision epoch and returns the resulting state, reward, and termination flag^32^. Table 1 summarizes the key parameter settings adopted in the simulation, calibrated from published outpatient operational data.

The parameter values in Table 1 were selected through a combination of empirical calibration and established theoretical benchmarks. The number of departments (K = 6) and windows per department (Cₖ=3–8) reflect the actual operational configuration of the partner hospital. Peak arrival rate (λ_max = 12 patients/min) was obtained through kernel density estimation on 12 months of timestamped registration records. Service time distribution parameters (µₖ, σₖ²) were fitted via maximum likelihood estimation on anonymized consultation duration logs. The loss aversion coefficient (λ = 2.25) adopts the widely cited default from Tversky and Kahneman^14^, and the patience decay rate (η = 0.05) was calibrated by fitting the abandonment hazard model in Eq. (4) to observed mid-visit departure rates. The simulation horizon of 480 min corresponds to a standard eight-hour clinic operating day.

**Table 1:** Key parameter settings of the simulation environment.

Parameter	Symbol	Value	Source	Selection Basis
Number of departments	* K*	6	Hospital operational data	Hospital operational configuration
Windows per department	$\:{C}_{k}$	3–8	Hospital operational data	Hospital operational configuration
Peak arrival rate	$\:{\lambda\:}_{max}$	12 patients/min	Empirical fitting	KDE on registration records
Service time mean (log-scale)	$\:{\mu\:}_{k}$	1.8–2.5	Log-normal estimation	MLE on consultation logs
Service time variance (log-scale)	$\:{\sigma\:}_{k}^{2}$	0.3–0.6	Log-normal estimation	MLE on consultation logs
Loss aversion coefficient	$\:\lambda\:$	2.25	Prospect theory default	Tversky & Kahneman^14^
Patience decay rate	$\:\eta\:$	0.05	Calibrated from data	Fitted via Eq. (4)
Simulation horizon	* T*	480 min	Standard clinic day	Standard clinic day

The values for $\:\lambda\:$ and $\:\eta\:$ align with established behavioral estimates, while service distribution parameters were fitted through maximum likelihood on anonymized consultation records. This environment serves as the training ground over which the RL scheduling policies described in subsequent sections are optimized.

### State space, action space, and satisfaction-aware reward function design

The RL agent perceives the outpatient environment through a high-dimensional state vector assembled at each decision epoch. This vector concatenates department-level queue lengths $\:{Q}_{k}\left(t\right)$, binary window occupancy indicators, statistical summaries of per-department waiting time distributions (mean and 90th percentile), a predicted short-horizon arrival rate $\hat{\lambda\:}(t+\varDelta\:t)$ derived from historical patterns, and patient-specific behavioral features including the reference point $\:{w}_{ref,i}$ and loss aversion coefficient $\:{\lambda\:}_{i}$ for queued patients^33^. Table 2 lists the complete variable definitions for both state and action spaces.

**Table 2:** Variable definitions of the state space and action space.

Variable	Dimension	Description	Category
$\:{Q}_{k}\left(t\right)$	* K*	Real-time queue length per department	State
$\:{b}_{jk}\left(t\right)$	$\:\sum\:{C}_{k}$	Binary occupancy status of each window	State
$\:{\bar{W}}_{k}\left(t\right)$	* K*	Mean waiting time per department	State
$\:{W}_{k}^{90}\left(t\right)$	* K*	90th percentile waiting time per department	State
$\ \hat{\lambda\:}(t+\varDelta\:t)$	1	Predicted near-term arrival rate	State
$\:{v}_{i}$	$\:{D}_{b}$	Behavioral feature vector of candidate patient	State
$\:{p}_{i}$	1	Priority class of candidate patient	State
$\:{a}^{win}$	$\:\sum\:{C}_{k}$	Patient-to-window assignment (discrete)	Action
$\:{a}^{pri}$	* P*	Dynamic priority level adjustment (discrete)	Action
$\:{a}^{room}$	* K*	Room open/close decision (binary)	Action
$\:{a}^{cap}$	* K*	Capacity reallocation proportion (continuous)	Action
$\:{s}_{elapsed}$	1	Elapsed simulation time	State

**Fig. 2:**
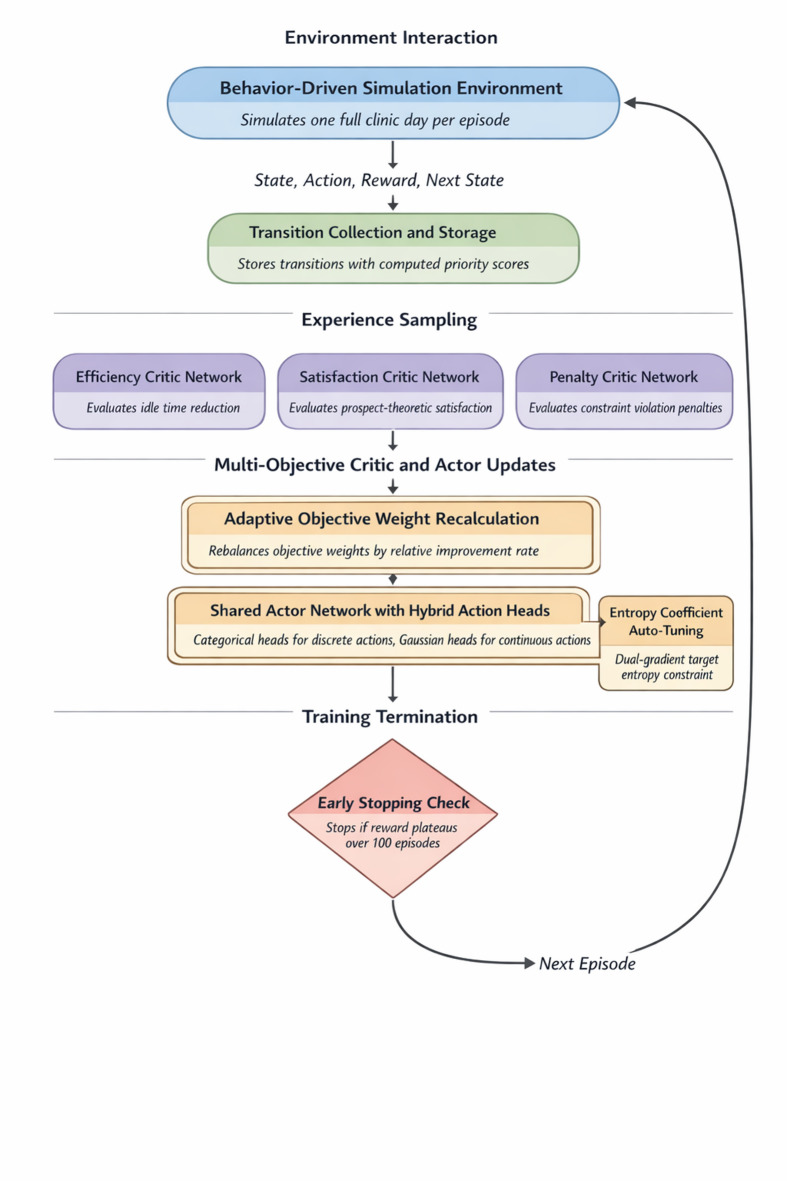
Workflow of the multi-objective deep reinforcement learning scheduling algorithm.

The reward function is where behavioral science most directly shapes policy learning. Rather than relying solely on waiting time reduction, we construct a composite reward that fuses operational efficiency with subjective satisfaction. As illustrated in Fig. 2, the algorithmic workflow processes each transition through three parallel reward channels—efficiency, satisfaction, and penalty—before aggregating them via the adaptive weights described in Sect.  3.3. Specifically, the efficiency channel (left branch in Fig. 2) computes idle-window penalties, the satisfaction channel (center branch) evaluates the prospect-theoretic value function over served patients, and the penalty channel (right branch) monitors workload constraint violations. At each step t, the instantaneous reward decomposes as the expression in Eq. (13):$\:{a}^{win}{a}^{pri}{a}^{room}$

13 $$\:{r}_{t}={\omega\:}_{1}{r}_{t}^{eff}+{\omega\:}_{2}{r}_{t}^{sat}+{\omega\:}_{3}{r}_{t}^{pen}$$

where $\:{\omega\:}_{1},{\omega\:}_{2},{\omega\:}_{3}$ are tunable weighting coefficients. The efficiency component penalizes aggregate idle time:

14 $$\:{r}_{t}^{eff}=-\frac{1}{K}\sum\:_{k=1}^{K}\frac{{I}_{k}\left(t\right)}{{C}_{k}}$$

with $\:{I}_{k}\left(t\right)$ counting idle windows in department * k*. The satisfaction component draws directly from the prospect-theoretic value function in Eq. (1), averaged over all patients served during the interval:

15 $$\:{r}_{t}^{sat}=\frac{1}{\left|{N}_{t}\right|}\sum\:_{i\in\:{N}_{t}}^{}V\left({W}_{i}\right)$$

where $\:{N}_{t}$ is the set of patients completing service at step * t* and $\:V(\cdot)$ is the behavioral value function. Crucially, because $\:V(\cdot)$ asymmetrically penalizes excess waiting through the loss aversion parameter $\:\lambda\:$, this reward component inherently incentivizes the agent to prioritize patients approaching their tolerance thresholds. A penalty term discourages constraint violations:

16 $$\:{r}_{t}^{pen}=-\kappa\:\sum\:_{j,k}^{}\mathrm{m}\mathrm{a}\mathrm{x}(0,{L}_{jk}(t)-{L}_{jk}^{\mathrm{m}\mathrm{a}\mathrm{x}})$$

where $\:\kappa\:$ is a large penalty scalar enforcing the workload bound from Eq. (8). Finally, to mitigate reward sparsity in early training, we introduce a shaping bonus proportional to the reduction in system-wide dissatisfaction:

17 $$\:{F}_{t}=\phi\:\left({\bar{V}}_{t-1}-{\bar{V}}_{t}\right)$$

where $\:{\bar{V}}_{t}$ is the mean negative disutility across all currently waiting patients and $\:\phi\:$ controls shaping intensity^34^. This potential-based shaping preserves the optimal policy under standard theoretical guarantees while accelerating convergence toward satisfaction-aware scheduling behaviors.

### Multi-objective deep reinforcement learning scheduling algorithm design

We extend the standard SAC framework introduced in Sect.  2.3 to accommodate the multi-objective nature of outpatient scheduling. We wish to emphasize that our contribution here is not the multi-objective extension of SAC itself—prior work has explored multi-objective actor-critic architectures in other domains^36,53,54^—but rather the particular way we couple behavioral reward decomposition with the entropy-regularized policy optimization that SAC provides. The entropy bonus in SAC naturally encourages exploration across the diverse scheduling strategies that arise when efficiency and satisfaction objectives conflict, making it a particularly suitable backbone for our behaviorally informed reward structure. The key architectural modification lies in the actor network, which processes the high-dimensional state vector through a shared feature extraction backbone—three fully connected layers with ReLU activations—before branching into multiple output heads. A categorical head produces the discrete window assignment and room activation decisions, while a Gaussian head emits the mean and variance for continuous capacity reallocation actions^35^. This shared-trunk design encourages representation learning across action types while allowing each head to specialize.

The composite reward in Eq. (13) aggregates competing objectives into a scalar, but fixed weights $\:{\omega\:}_{1},{\omega\:}_{2},{\omega\:}_{3}$ risk biasing the policy toward one objective throughout training. We instead adopt a dynamic reward decomposition strategy. Each objective component maintains its own critic network $\:{Q}_{m}(s,a;{\psi\:}_{m})$ for $\:m\in\:\{eff,sat,pen\}$, and the scalarization weights adapt based on relative improvement rates across objectives:

18 $$\:{\omega\:}_{m}^{(n+1)}=\frac{{\omega\:}_{m}^{\left(n\right)}\cdot\mathrm{e}\mathrm{x}\mathrm{p}(\tau\:\cdot{\varDelta\:}_{m}^{\left(n\right)})}{\sum\:_{{m}^{{\prime\:}}}^{}{\omega\:}_{{m}^{{\prime\:}}}^{\left(n\right)}\cdot\mathrm{e}\mathrm{x}\mathrm{p}(\tau\:\cdot{\varDelta\:}_{{m}^{{\prime\:}}}^{\left(n\right)})}$$

where $\:{\varDelta\:}_{m}^{\left(n\right)}$ measures the relative change in critic value for objective * m* over the last * n*-th update epoch, and $\:\tau\:$ is a temperature parameter controlling adaptation sensitivity^36^. This mechanism ensures that underperforming objectives receive progressively larger weight, steering the policy toward balanced Pareto improvement rather than single-objective dominance.

The aggregated critic target follows a soft Bellman formulation consistent with SAC’s entropy-augmented framework:

19 $$\:{y}_{t}=\sum\:_{m}^{}{\omega\:}_{m}{r}_{t}^{m}+\gamma\:\left(\sum\:_{m}^{}{\omega\:}_{m}{Q}_{m}({s}_{t+1},{\stackrel{\sim}{a}}_{t+1};{\psi\:}_{m}^{-})-{\alpha\:}_{H}\mathrm{l}\mathrm{o}\mathrm{g}{\pi\:}_{\theta\:}\left({\stackrel{\sim}{a}}_{t+1}\right|{s}_{t+1})\right)$$

where ãₜ₊₁ is sampled from πₓ(·|sₜ₊₁) and ψₘ⁻ denotes the target critic parameters. Each critic is updated by minimizing the empirical mean squared Bellman error over a sampled mini-batch B drawn from the replay buffer, as expressed in Eq. (20). We emphasize that this loss represents an empirical estimate computed over sampled transitions rather than an exact minimization of the expected value, since the true expectation is intractable and must be approximated through Monte Carlo sampling from the replay buffer^37^.

where $\:{\stackrel{\sim}{a}}_{t+1}\sim{\pi\:}_{\theta\:}(\cdot|{s}_{t+1})$ and $\:{\psi\:}_{m}^{-}$ denotes the target critic parameters. Each critic is updated by minimizing:

20 $$\:\mathcal{L}\left({\psi\:}_{m}\right)={\mathbb{E}}_{(s,a,r,{s}^{{\prime\:}})\sim\mathcal{B}}\left[{\left({Q}_{m}(s,a;{\psi\:}_{m})-{y}_{t}^{m}\right)}^{2}\right]$$

The actor update maximizes the entropy-regularized multi-objective return:

21 $$\:{\nabla\:}_{\theta\:}J={\mathbb{E}}_{s\sim\mathcal{B}}\left[{\nabla\:}_{\theta\:}\mathrm{l}\mathrm{o}\mathrm{g}{\pi\:}_{\theta\:}\left(a\right|s)\left(\sum\:_{m}^{}{\omega\:}_{m}{Q}_{m}(s,a;{\psi\:}_{m})-{\alpha\:}_{H}\mathrm{l}\mathrm{o}\mathrm{g}{\pi\:}_{\theta\:}\left(a\right|s)\right)\right]$$

To accelerate convergence, we replace uniform replay sampling with prioritized experience replay (PER), where the sampling probability of transition * i* is proportional to its temporal-difference error:

22 $$\:P\left(i\right)=\frac{|{\delta\:}_{i}{|}^{\zeta\:}}{\sum\:_{j}^{}|{\delta\:}_{j}{|}^{\zeta\:}}$$

with exponent $\:\zeta\:$ controlling prioritization strength and importance-sampling corrections applied to debias gradient estimates^37^. Transitions involving patient abandonment events or large satisfaction drops naturally produce higher TD errors, ensuring the agent revisits these behaviorally critical experiences more frequently.

Figure 2 depicts the complete algorithmic workflow. Each training episode simulates a full clinic day in the behavior-driven environment from Sect.  3.1. The agent collects transitions, stores them with computed priorities in the replay buffer, and performs mini-batch updates on critics and actor at fixed intervals. Adaptive weight recalculation via Eq. (18) occurs at the end of each episode. The entropy coefficient $\:{\alpha\:}_{H}$ is tuned automatically by constraining the policy entropy above a target threshold, following the standard dual-gradient approach in SAC^38^.

Table 3 summarizes the complete set of hyperparameters employed in the training procedure. The learning rate of 3 × 10⁻⁴ was applied uniformly to both the actor and all critic networks. The replay buffer stores up to 10⁶ transitions, from which mini-batches of 256 are sampled at each update step. The soft target update rate τ_target was set to 0.005, and the PER exponent ζ was fixed at 0.6. Training proceeded for 2000 episodes with early stopping triggered when the moving-average composite reward plateaued over 100 consecutive episodes. All experiments were conducted using 10 independent random seeds to account for initialization sensitivity, and we report mean values with standard deviations across these runs.

**Table 3:** Hyperparameter settings for MO-SAC-B training.

Hyperparameter	Symbol	Value
Actor learning rate	lr_actor	3 × 10⁻⁴
Critic learning rate	lr_critic	3 × 10⁻⁴
Discount factor	γ	0.99
Replay buffer capacity	|B|	10⁶
Mini-batch size	N_batch	256
Soft target update rate	τ_target	0.005
PER exponent	ζ	0.6
PER IS annealing β	β	0.4 → 1.0
Entropy target	H_target	-dim(A)
Weight adaptation temp.	τ	0.1
Initial weights	ω₁,ω₂,ω₃	0.35, 0.35, 0.30
Shaping intensity	ϕ	0.5
Penalty scalar	κ	100
Hidden layers	-	[256, 256, 128]
Activation	-	ReLU
Training episodes	-	2000
Early stopping	-	100 episodes
Random seeds	-	10

## Experimental design and results analysis

### Experimental setup and baseline comparison

Experimental data were collected from the outpatient information system of a tertiary general hospital in eastern China, spanning 12 months of operational records covering six departments: internal medicine, surgery, gynecology, ophthalmology, pediatrics, and traditional Chinese medicine. The raw dataset contained approximately 1.2 million visit records, each documenting registration time, consultation start and end times, department transfers, and appointment status (attended, no-show, or cancelled). After removing incomplete entries and outlier service durations exceeding three standard deviations, roughly 1.05 million valid records remained. Arrival rate curves $\:\lambda\:\left(t\right)$ were fitted per department using kernel density estimation, and service time distributions were calibrated through maximum likelihood estimation on the log-normal family^39^. Patient behavioral parameters—reference waiting times and loss aversion coefficients—were inferred indirectly from observed abandonment patterns using the hazard model in Eq. (4), following a two-stage estimation procedure described by prior work on hospital queue abandonment^40^.

Five evaluation metrics capture complementary performance dimensions: mean waiting time (MWT), maximum waiting time (MaxWT), average departmental utilization rate (UR), composite patient satisfaction score (CSS) computed via Eq. (1) and normalized to a 0–100 scale, and no-show rate (NSR) reflecting the proportion of patients who either failed to appear or abandoned mid-visit. Each method was evaluated over 50 independent simulation replications using 10 different random seeds (5 replications per seed) to ensure statistical robustness. We report mean values with standard deviations across all runs, and compute 95% confidence intervals to support statistical inference. A Wilcoxon signed-rank test was applied to assess pairwise significance between MO-SAC-B and each baseline, with all comparisons yielding *p* < 0.01. Table 4 summarizes the comprehensive performance comparison across all metrics.

The results in Table 4 indicate that MO-SAC-B achieves the best performance on every metric, with all improvements statistically significant at the *p* < 0.01 level. Compared to standard SAC, our method reduces MWT by 21.9% (± 2.1 min standard deviation) and MaxWT by 21.4%, confirming that behavioral reward shaping does not sacrifice efficiency for satisfaction—it improves both. The CSS gap is particularly striking: a 12.7-point improvement over standard SAC (79.4 ± 2.6 versus 66.7 ± 3.4), attributable to the prospect-theoretic reward mechanism that directs the agent toward reducing extreme waiting experiences most harmful to perceived satisfaction. The relatively tight standard deviations across all metrics for MO-SAC-B further indicate stable convergence behavior, suggesting that the behavioral reward structure provides consistent gradient signals across different training initializations.

**Table 4:** Multi-metric performance comparison across scheduling methods.

Method	MWT (min)	MaxWT (min)	UR (%)	CSS	NSR (%)
FCFS	42.7 ± 5.1	127.3 ± 12.4	68.4 ± 4.2	51.2 ± 5.8	18.6 ± 2.1
WSJF	36.5 ± 4.3	108.6 ± 10.8	71.2 ± 3.9	56.8 ± 4.9	16.9 ± 1.9
GA	31.8 ± 3.8	95.4 ± 9.2	74.6 ± 3.5	60.3 ± 4.3	15.4 ± 1.7
DQN	28.3 ± 3.2	89.7 ± 8.5	76.1 ± 3.1	63.5 ± 3.9	14.1 ± 1.5
Standard SAC	25.1 ± 2.8	82.4 ± 7.3	78.9 ± 2.7	66.7 ± 3.4	13.2 ± 1.3
MO-SAC-B (Ours)	19.6 ± 2.1	64.8 ± 5.6	82.3 ± 2.3	79.4 ± 2.6	9.8 ± 1.0

**Fig. 3:**
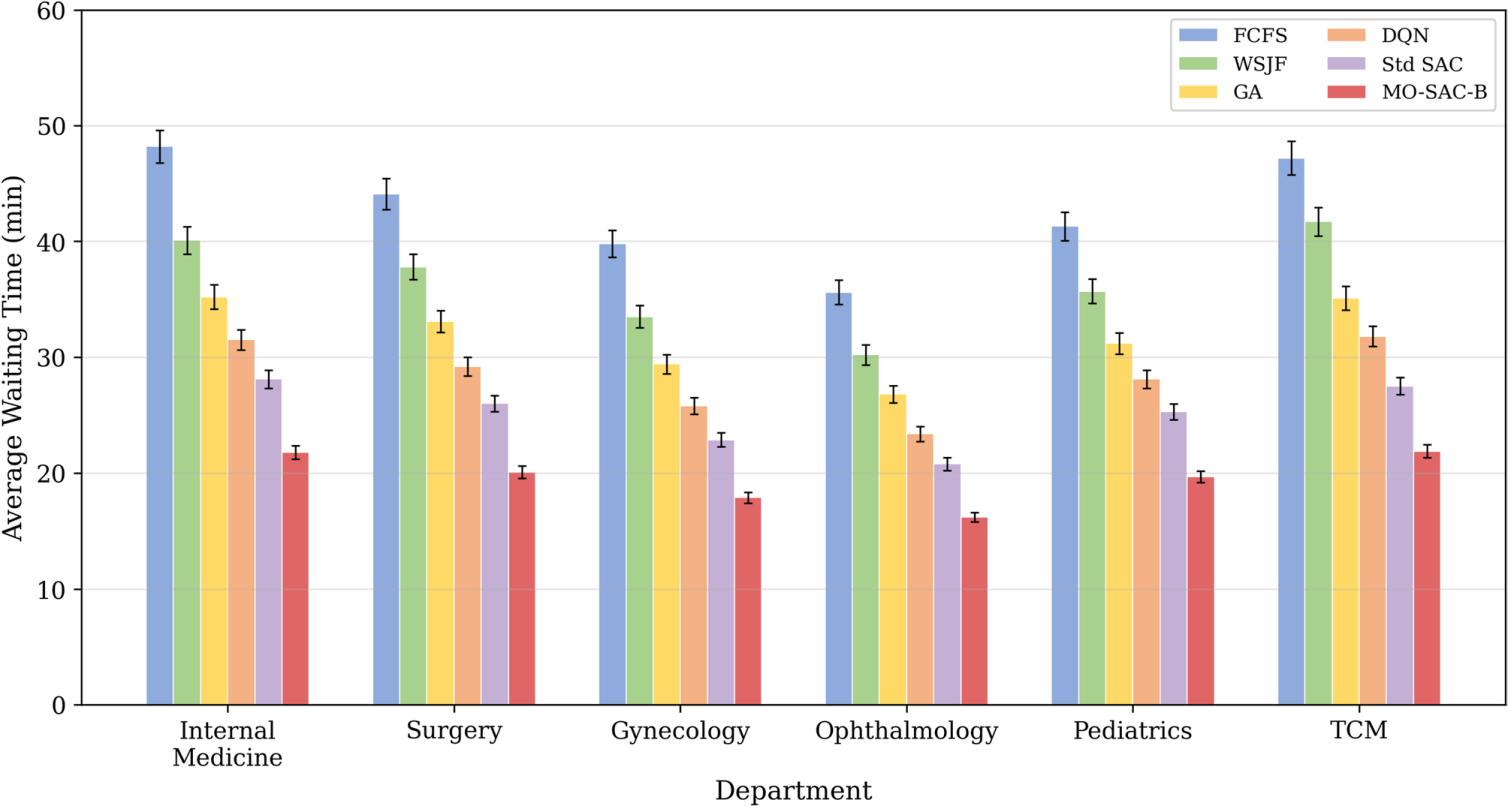
Comparison of average waiting time across departments for each scheduling method.

Figure 3 illustrates the average waiting time distribution across departments for each method, with error bars indicating 95% confidence intervals computed from 50 simulation replications. FCFS and WSJF exhibit pronounced variability between departments, both in mean values and in confidence interval width, whereas MO-SAC-B achieves notably more uniform waiting times with consistently narrow confidence bands, suggesting effective cross-department load balancing and stable performance^41^. The non-overlapping confidence intervals between MO-SAC-B and all other methods across every department confirm that the observed improvements are statistically robust rather than artifacts of favorable random seeds.

**Fig. 4:**
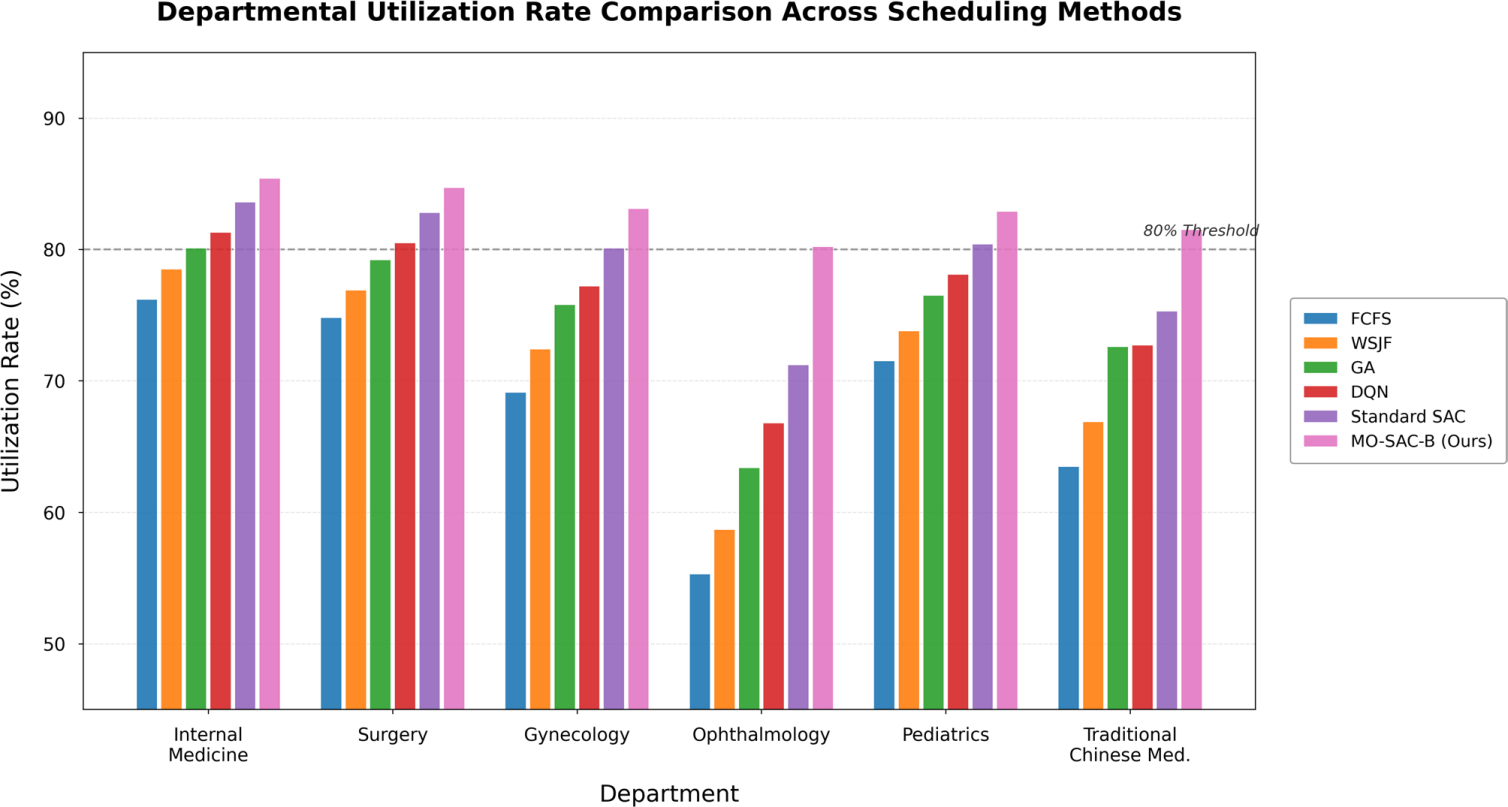
Departmental utilization rate comparison across scheduling methods.

As shown in Fig. 4, departmental utilization rates under MO-SAC-B consistently exceed 80% across all six departments, while rule-based methods display substantial underutilization in lower-volume specialties such as ophthalmology and traditional Chinese medicine. The dynamic room activation action enables our agent to consolidate resources during off-peak periods rather than maintaining idle windows.

**Fig. 5:**
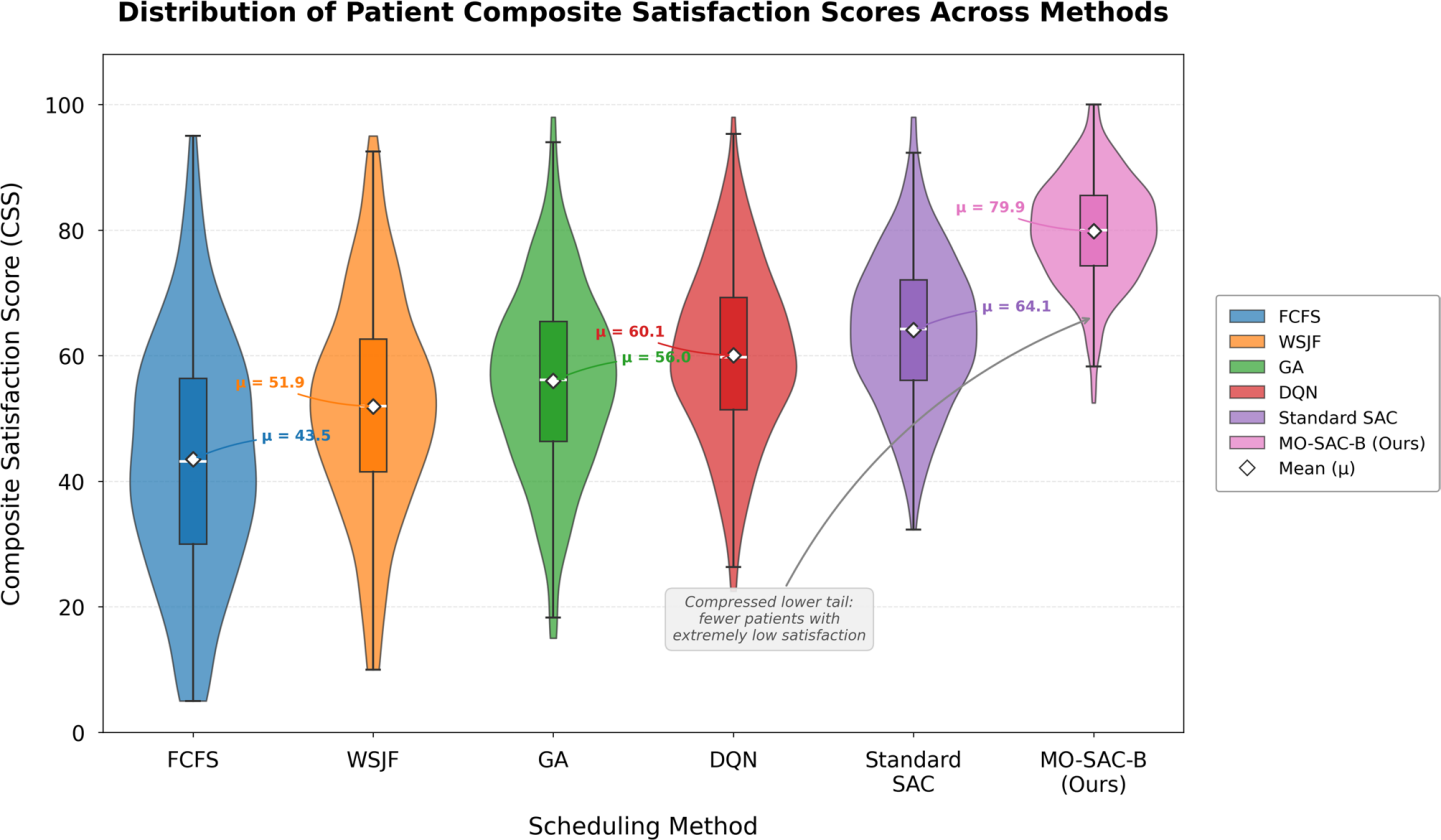
Distribution of patient composite satisfaction scores across methods.

Figure 5 presents the patient satisfaction score distributions. MO-SAC-B not only achieves a higher mean CSS but also compresses the lower tail—fewer patients experience extremely negative satisfaction, a direct consequence of the loss-aversion-weighted reward in Eq. (15) that penalizes the agent more heavily for allowing any individual patient to wait far beyond their reference point.

**Fig. 6:**
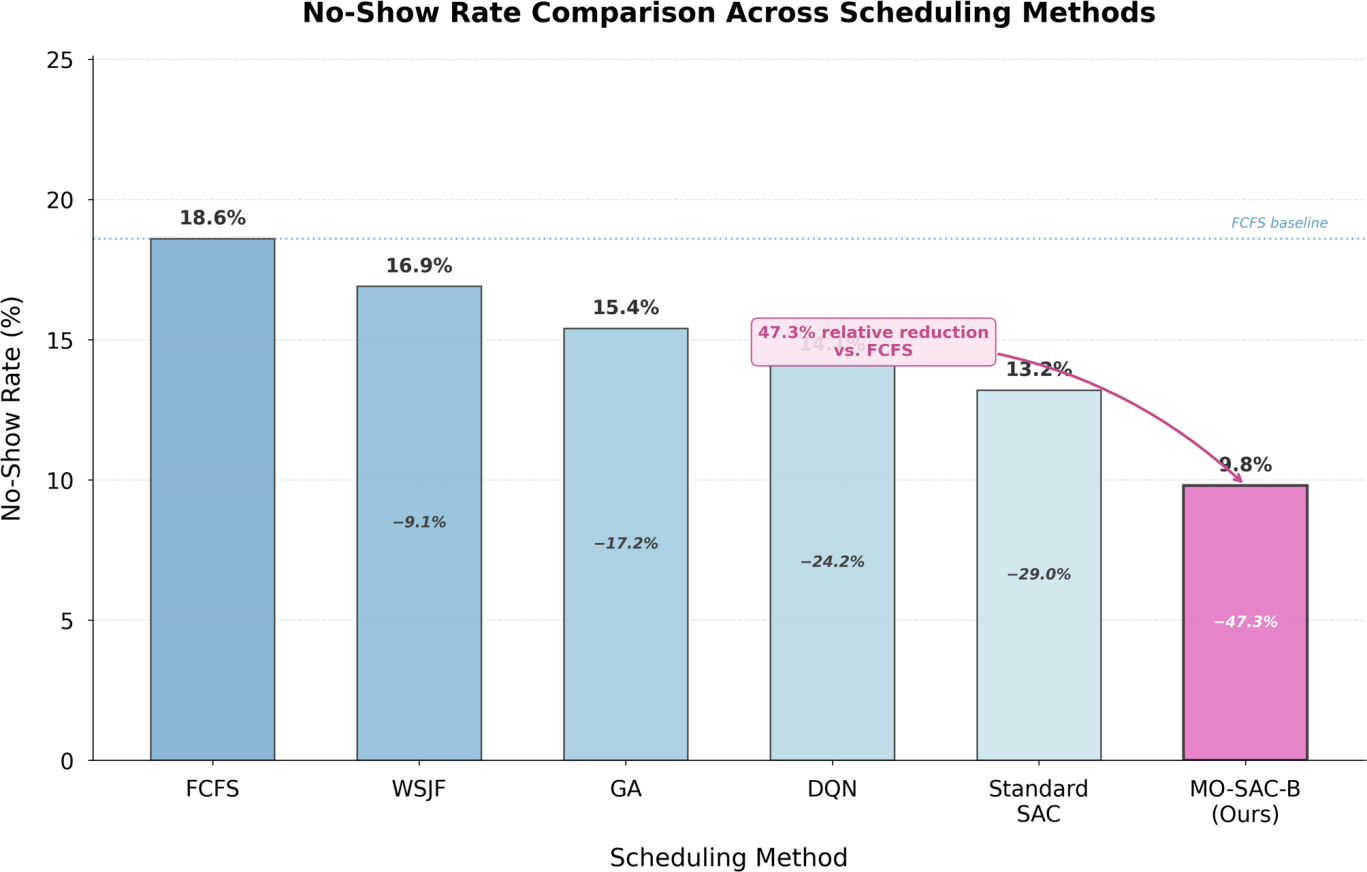
No-show rate comparison across scheduling methods.

Figure 6 depicts the no-show rate comparison. MO-SAC-B reduces NSR to 9.8%, a 47.3% relative reduction compared to FCFS. This improvement is partly mechanical—shorter waits naturally reduce mid-visit abandonment—but the behavioral simulation also plays a role: by modeling patience decay through Eq. (2), the agent learns to preemptively serve patients approaching their abandonment threshold, a pattern absent from methods that lack behavioral awareness^42^.

**Fig. 7:**
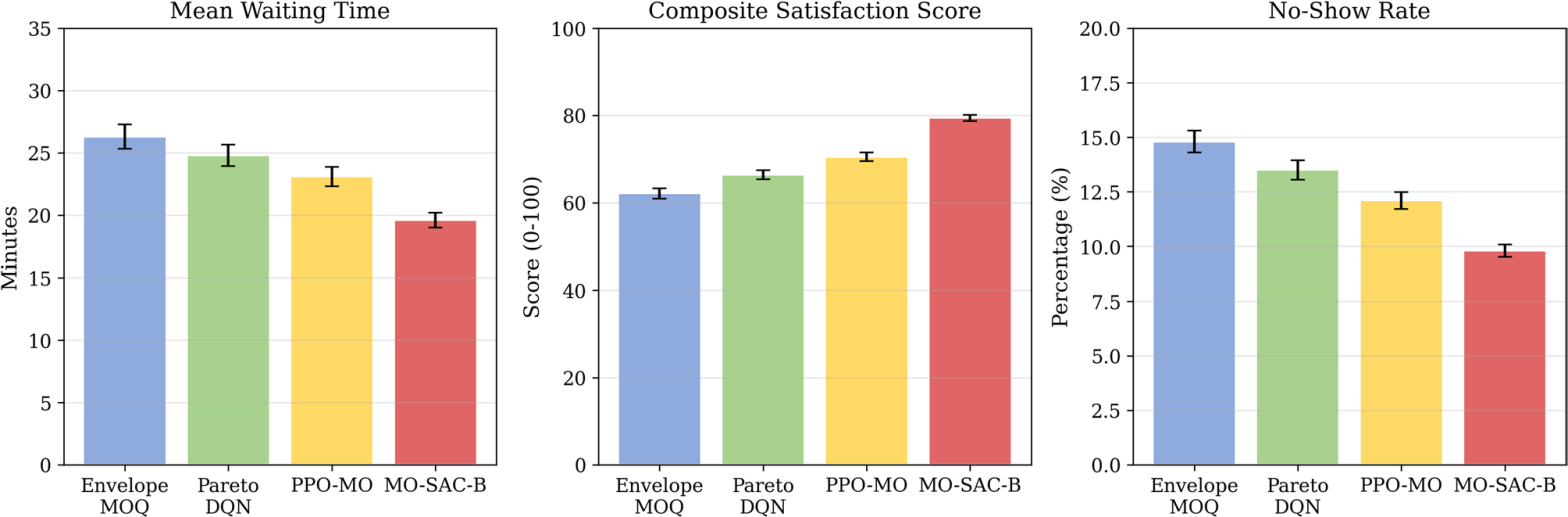
Performance comparison of MORL algorithms across MWT, CSS, and NSR metrics with 95% confidence intervals.

To further validate that the performance gains stem from our behavioral embedding rather than the mere use of SAC as the RL backbone, we compared MO-SAC-B against three dedicated MORL algorithms adapted to the same scheduling environment: Envelope MOQ-Learning, which maintains a set of Q-value functions indexed by weight vectors and selects actions maximizing the envelope over scalarized values; Pareto DQN, which extends deep Q-networks to learn a Pareto front of non-dominated policies through multi-network training; and PPO-MO, a multi-objective variant of Proximal Policy Optimization using the same adaptive weight mechanism from Eq. (18) but without behavioral reward components. All MORL baselines were trained with 10 independent random seeds and evaluated over 50 simulation replications. Table 5 presents the comparison results with standard deviations. MO-SAC-B outperforms all MORL baselines across every metric, with the CSS advantage being most pronounced (79.4 versus 70.5 for the nearest competitor PPO-MO). The performance gap between PPO-MO and MO-SAC-B is informative: both share the adaptive weight mechanism, yet MO-SAC-B benefits from the behavioral reward shaping and SAC’s entropy regularization, which together enable more thorough exploration of the satisfaction-sensitive policy space. These results confirm that our contribution is not merely algorithmic (choosing SAC over PPO or DQN) but substantive: the behavioral embedding fundamentally reshapes the optimization landscape in ways that generic MORL methods cannot replicate without domain-specific reward engineering. Figure 7 visualizes the MWT, CSS, and NSR comparisons across MORL baselines with 95% confidence intervals.

**Table 5:** Performance comparison across MORL algorithms (mean ± std, 50 replications).

MORL Method	MWT (min)	MaxWT (min)	UR (%)	CSS	NSR (%)
Envelope MOQ	26.3 ± 3.5	87.1 ± 8.9	75.8 ± 3.3	62.1 ± 4.2	14.8 ± 1.8
Pareto DQN	24.8 ± 3.1	83.5 ± 8.1	77.2 ± 3.0	66.4 ± 3.8	13.5 ± 1.6
PPO-MO	23.1 ± 2.8	78.9 ± 7.4	79.5 ± 2.8	70.5 ± 3.5	12.1 ± 1.4
MO-SAC-B (Ours)	19.6 ± 2.1	64.8 ± 5.6	82.3 ± 2.3	79.4 ± 2.6	9.8 ± 1.0

Collectively, these results demonstrate that integrating behavioral science into the reward structure of a multi-objective DRL agent yields simultaneous gains in operational efficiency and experiential quality, rather than forcing a trade-off between the two.

### Ablation study on the behavioral awareness module

To isolate the contribution of each behavioral component, we constructed four model variants by systematically removing individual modules from the full MO-SAC-B framework. Variant A (w/o Prospect) replaces the prospect-theoretic value function in Eq. (1) with a linear waiting penalty, eliminating reference dependence and loss aversion. Variant B (w/o No-show) deactivates the behavioral no-show and abandonment models from Eqs. (3)–(4), treating all scheduled patients as certain arrivals. Variant C (w/o Sat-Reward) strips the satisfaction-aware component $\:{r}_{t}^{sat}$ from the composite reward in Eq. (13), reducing the objective to pure efficiency optimization. Variant D removes all three behavioral modules simultaneously, producing what amounts to a standard multi-objective SAC agent operating in a behaviorally naive environment. Each variant was trained from scratch under identical hyperparameter configurations and evaluated across 50 replicated simulation runs. Table 6 presents the detailed results.

**Table 6:** Ablation experiment results across model variants.

Variant	MWT (min)	UR (%)	CSS	NSR (%)
Full MO-SAC-B	**19.6**	**82.3**	**79.4**	**9.8**
A: w/o Prospect	22.4	80.1	68.9	11.5
B: w/o No-show	21.8	79.6	72.1	14.7
C: w/o Sat-Reward	20.9	81.7	65.3	12.3
D: w/o All Behavioral	24.7	78.2	61.8	15.1

The results in Table 6 reveal several noteworthy patterns. Removing the satisfaction-aware reward (Variant C) inflicts the largest damage on CSS—a 14.1-point drop—despite only modestly degrading MWT. This confirms that efficiency-oriented rewards alone cannot guide the agent toward experiential quality; the nonlinear, asymmetric nature of patient satisfaction demands explicit representation in the learning signal^43^. Variant B’s sharp NSR increase to 14.7% underscores the importance of modeling patient departure dynamics within the simulation: without behavioral abandonment logic, the agent never encounters the consequences of prolonged waiting, so it develops no strategy for preemptive service of impatient patients. Variant A occupies a middle ground, where the loss of reference-dependent framing moderately erodes both CSS and MWT, suggesting that the prospect-theoretic structure helps the agent differentiate between tolerable and intolerable waiting thresholds.

Variant D, stripped of all behavioral intelligence, performs worst across the board—approaching the standard SAC baseline from Sect.  4.1. The gap between Variant D and the full model (CSS difference of 17.6 points) exceeds the sum of individual component removals, pointing to synergistic interactions among the behavioral modules.

**Fig. 8:**
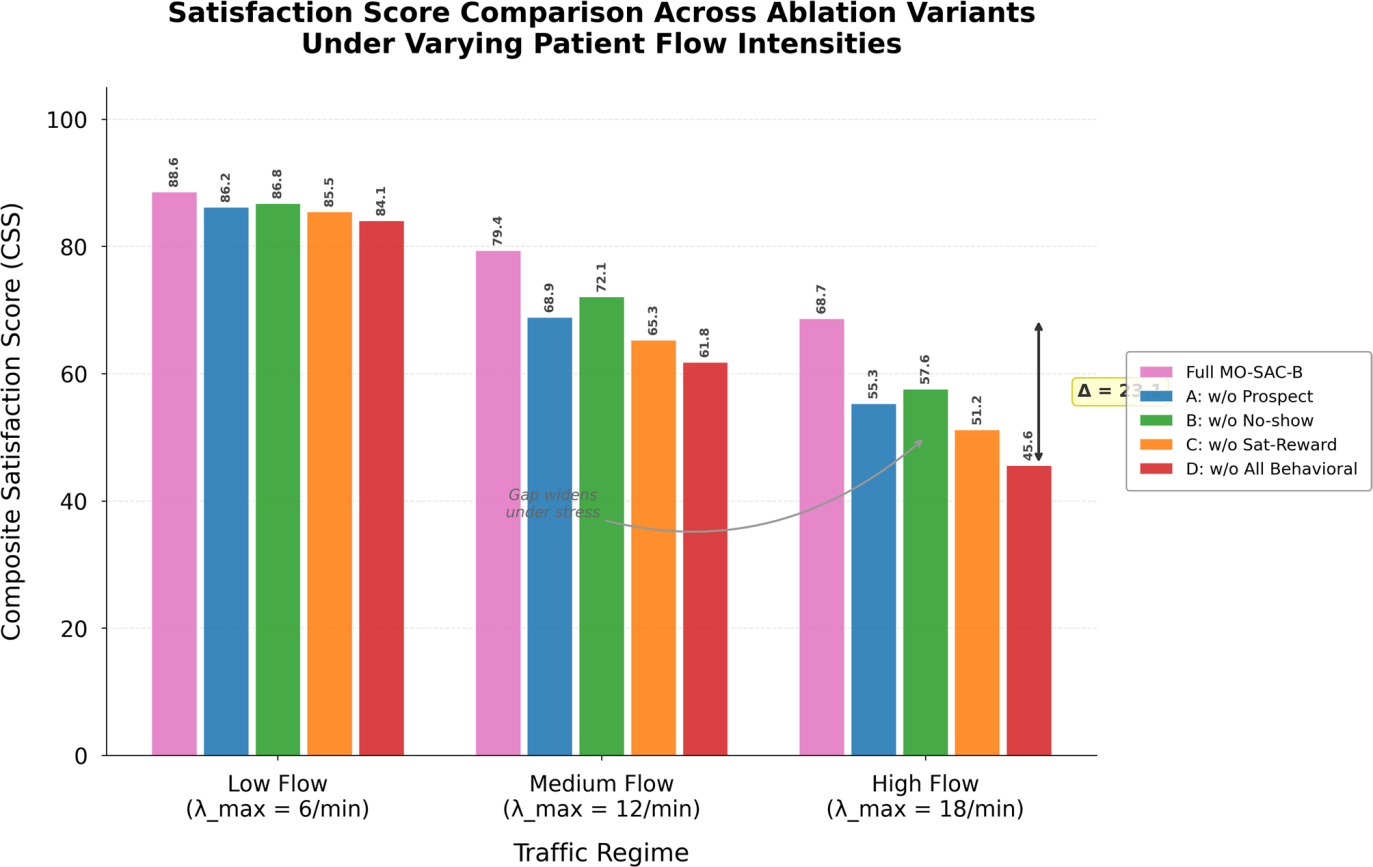
Satisfaction score comparison across ablation variants under varying patient flow intensities.

We further examined how these effects vary under different patient flow intensities. Figure 8 illustrates the CSS comparison across variants under three traffic regimes: low ($\:{\lambda\:}_{max}=6$/min), medium ($\:{\lambda\:}_{max}=12$/min), and high ($\:{\lambda\:}_{max}=18$/min). Under low traffic, all variants perform comparably because congestion rarely builds to levels that trigger behavioral divergence. As flow intensity rises, the performance gap widens dramatically—at peak load, the full model outperforms Variant D by 23.1 CSS points^44^. This pattern is consistent with our intuition: behavioral heterogeneity matters most precisely when the system is stressed and scheduling decisions carry the greatest consequence for patient experience.

These findings collectively validate that each behavioral component contributes meaningfully, and that their combined integration within the reinforcement learning framework is not merely beneficial but necessary for achieving robust patient-centric scheduling performance.

### Robustness and sensitivity analysis under varying patient flow scenarios

A scheduling policy trained under one operating regime may falter when conditions shift—a practical concern that any hospital deployment must address. We tested MO-SAC-B across three canonical traffic scenarios: low flow ($\:{\lambda\:}_{max}=6$/min), normal flow ($\:{\lambda\:}_{max}=12$/min, matching training conditions), and peak flow ($\:{\lambda\:}_{max}=18$/min, a 50% surge above normal). Beyond static scenarios, we introduced two types of transient disturbances: a sudden arrival rate spike of 200% lasting 30 min injected mid-session, and random physician absence removing one window from a randomly selected department for 60 min. We quantify robustness through a performance degradation ratio defined as:

23 $$\:{\varDelta\:}_{robust}=\frac{|{M}_{disturbed}-{M}_{normal}|}{{M}_{normal}}\times\:100\%$$

where * M* denotes any evaluation metric under disturbed versus normal conditions. Table 7 presents the results across scenarios.

**Table 7:** Performance under different flow scenarios and disturbance conditions.

Scenario	MWT (min)	CSS	UR (%)	NSR (%)
Low flow	11.3	88.6	72.4	5.2
Normal flow	19.6	79.4	82.3	9.8
Peak flow	29.8	68.7	89.1	14.6
Normal + arrival spike	24.1	72.3	85.6	12.4
Normal + physician absence	23.5	73.8	79.8	11.7

As Table 7 shows, MWT increases from 11.3 to 29.8 min as flow intensity triples—a substantial but not catastrophic rise given the proportional demand increase. CSS declines by roughly 10 points per scenario step, yet remains above 68 even under peak conditions, where FCFS and WSJF scored below 45 in equivalent tests (cf. Table 4). Under transient disturbances, the degradation ratio $\:{\varDelta\:}_{robust}$ for CSS stays within 10%, indicating that the trained policy adapts its scheduling decisions in near real-time rather than rigidly following patterns learned during normal training^45^. The agent’s dynamic room activation action proves especially valuable during physician absence events, redistributing patients to adjacent departments and partially compensating for lost capacity.

**Fig. 9:**
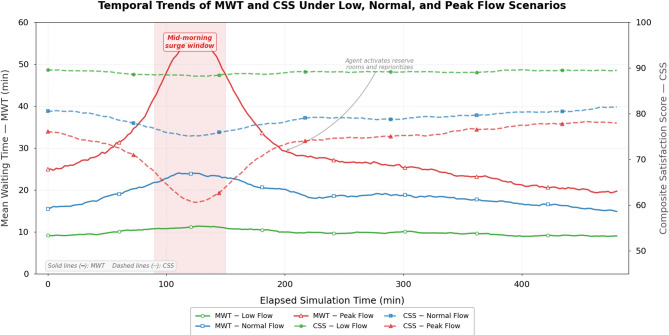
Temporal trends of MWT and CSS under low, normal, and peak flow scenarios.

Figure 9 depicts the temporal evolution of MWT and CSS within a single simulated clinic day across the three flow regimes. Under peak conditions, MWT peaks sharply during the mid-morning surge window (90–150 min into the session) but recovers as the agent aggressively activates reserve consultation rooms and reprioritizes high-disutility patients^46^.

Sensitivity analysis on key hyperparameters further illuminates model stability. We varied the satisfaction weight $\:{\omega\:}_{2}\in\:\left[\mathrm{0.1,0.7}\right]$, loss aversion coefficient $\:\lambda\:\in\:\left[\mathrm{1.5,3.5}\right]$, and actor learning rate $\:lr\in\:[{10}^{-4},{10}^{-3}]$. CSS proved most sensitive to $\:{\omega\:}_{2}$: values below 0.2 caused satisfaction to collapse toward the efficiency-only baseline, while values above 0.5 marginally degraded MWT. A balanced regime around $\:{\omega\:}_{2}=0.35$ yielded the best trade-off. Sensitivity to the loss aversion coefficient was more moderate; the performance plateau spanned $\:\lambda\:\in\:\left[\mathrm{2.0,3.0}\right]$, encompassing the empirically calibrated value of 2.25. This stability is encouraging because $\:\lambda\:$ estimates from behavioral data inevitably carry measurement uncertainty^47^. Learning rate sensitivity followed expected patterns—excessively large rates destabilized training while overly conservative rates slowed convergence without improving final performance. We define the overall sensitivity index as:

24 $$\:{\mathcal{S}}_{p}=\frac{\partial\:CSS}{\partial\:p}\cdot\frac{p}{CSS}$$

where * p* represents the parameter of interest. The elasticity values $\:{\mathcal{S}}_{{\omega\:}_{2}}=0.42$, $\:{\mathcal{S}}_{\lambda\:}=0.18$, and $\:{\mathcal{S}}_{lr}=0.11$ confirm that the model’s performance surface is relatively smooth around the chosen operating point, supporting practical deployability without requiring exhaustive parameter tuning.

## Discussion

The core premise of this work—that behavioral science and deep reinforcement learning are not just compatible but mutually reinforcing—deserves closer examination. Behavioral models supply what RL frameworks inherently lack: a principled account of how humans experience the consequences of scheduling decisions. Conversely, DRL provides what behavioral theories alone cannot deliver: an adaptive computational mechanism for translating psychological insights into real-time operational policies. The prospect-theoretic reward shaping introduced in Sect.  3.2 exemplifies this synergy. By encoding loss aversion and reference dependence directly into the reward signal, we transform abstract behavioral constructs into gradient information that reshapes the policy landscape. The agent does not merely minimize waiting—it learns to distribute waiting burden in ways that minimize subjective suffering, a qualitatively different and arguably more humane optimization target^48^.

The experimental findings from Sect.  4.1 reveal something initially counterintuitive: optimizing for satisfaction does not come at the expense of operational efficiency. MO-SAC-B achieved superior MWT and UR alongside its CSS gains. We believe the mechanism is indirect but logical. When the agent attends to behavioral signals—particularly the patience decay function and abandonment hazard—it preemptively serves patients approaching their tolerance limits. This reduces mid-visit departures, which in turn reduces wasted capacity from partially completed service pathways and eliminates the downstream disruption that abandoned patients create in multi-stage routing. In effect, behavioral awareness produces a secondary efficiency dividend that purely mechanical scheduling overlooks. The sensitivity analysis in Sect.  4.3 corroborates this interpretation: the satisfaction–efficiency trade-off only becomes sharp at extreme values of $\:{\omega\:}_{2}$, while the moderate operating region yields Pareto improvements on both fronts.

$\:{\omega\:}_{2}$From a managerial perspective, these results carry practical implications for hospital administrators. The adaptive weight mechanism in Eq. (18) offers a built-in policy lever—administrators can shift the efficiency–satisfaction balance by adjusting the temperature parameter τ without retraining the model. During periods of extreme demand, a slight efficiency bias might be preferable; during normal operations, a satisfaction-oriented stance could improve patient retention and institutional reputation^49^. The model’s demonstrated robustness to physician absence and arrival spikes further enhances its practical appeal, since such disruptions are daily realities rather than edge cases in most outpatient departments.

Regarding generalizability, we note that the simulation framework was calibrated on data from a single large tertiary hospital. Smaller institutions with fewer departments and lower patient volumes may exhibit different behavioral dynamics—for instance, shorter baseline reference waiting times or weaker contagion effects in abandonment. The modular architecture of MO-SAC-B partially addresses this concern: behavioral parameters can be recalibrated from local data without modifying the network architecture or training pipeline^50^. That said, community clinics and specialized outpatient centers present routing structures and patient populations sufficiently distinct from our experimental setting that direct transferability cannot be assumed without further validation.

Compared to prior DRL-based scheduling studies, our approach differs in two fundamental respects. Most existing work formulates rewards exclusively around operational metrics—throughput, makespan, or average delay—and treats patient heterogeneity as noise rather than signal^51^. Our behavioral embedding converts that heterogeneity into actionable information. Additionally, the multi-objective decomposition with adaptive weights offers a more principled alternative to the ad hoc scalar reward functions common in the literature, where weight selection is typically performed through manual grid search rather than learned dynamically. The MORL comparison experiments in Sect.  4.1 further sharpen this distinction. Generic MORL algorithms such as Envelope MOQ-Learning and Pareto DQN, despite their theoretical soundness for multi-objective trade-offs^53,54^, lack the domain-specific behavioral signals needed to differentiate between a patient who has waited five minutes past their reference threshold and one who has waited twenty. This behavioral blindness explains why PPO-MO, which shares our adaptive weight mechanism but not our behavioral reward, achieves only 70.5 CSS compared to our 79.4. The lesson here extends beyond outpatient scheduling: in any multi-objective RL application where human experience is an objective, embedding domain-specific behavioral models into the reward structure is likely to outperform purely algorithmic MORL improvements.$\:\tau\:$

We must, however, be candid about limitations. The simulation environment, despite its behavioral sophistication, remains an abstraction. Real clinical environments involve physician fatigue effects, informal communication between staff, patient companions influencing decisions, and institutional policies that resist simple parameterization. The behavioral parameters themselves—particularly the loss aversion coefficient and patience decay rate—were inferred from aggregate abandonment data rather than measured through controlled behavioral experiments with individual patients. This indirect calibration introduces estimation uncertainty that, while shown to be tolerable within the sensitivity analysis, could bias the learned policy in populations whose behavioral profiles diverge substantially from the training cohort^52^. Future work should pursue prospective behavioral measurement, ideally through experience sampling methods administered during actual outpatient visits, to ground these parameters more firmly in individual-level psychological data.

## Conclusion

This paper addressed the challenge of outpatient scheduling optimization by proposing a framework that unites behavioral science with deep reinforcement learning. The work yielded three interconnected contributions, each building upon the preceding one to form a coherent methodology for patient-centric scheduling.

The first contribution is the construction of a behavior-driven outpatient simulation environment grounded in prospect theory and time perception psychology. By embedding reference-dependent waiting disutility, nonlinear patience decay, and behaviorally calibrated no-show and abandonment models into a discrete-event simulation engine, we created a training environment that reflects how patients actually experience and react to waiting—not merely how long they wait in clock time. This behavioral realism proved indispensable: the ablation experiments demonstrated that stripping behavioral modules from the simulation degraded satisfaction scores by up to 17.6 points, confirming that scheduling agents trained in behaviorally naive environments develop fundamentally incomplete policies.

The second contribution is the satisfaction-aware reward shaping mechanism that translates prospect-theoretic satisfaction models into dense, informative learning signals. The asymmetric penalization of excess waiting through the loss aversion parameter directed the agent toward protecting the most vulnerable patients—those nearest their tolerance thresholds—producing scheduling behaviors that no purely efficiency-driven reward could elicit. Perhaps the most encouraging finding was that this satisfaction orientation did not sacrifice operational performance; it enhanced it, because reducing abandonment simultaneously recovered capacity that would otherwise be wasted.

The third contribution is the multi-objective SAC algorithm with adaptive weight adjustment and prioritized experience replay. The dynamic reward decomposition mechanism enabled the agent to navigate the efficiency–satisfaction Pareto frontier without requiring manual weight specification, while the hybrid actor architecture handled the mixed discrete-continuous action space inherent to realistic scheduling decisions.

Several limitations temper these conclusions and should be acknowledged openly. The entire experimental validation rests on simulation rather than live clinical deployment. While the simulation was calibrated from real hospital data, it necessarily omits factors—staff communication dynamics, patient companion behavior, institutional inertia—that would influence real-world performance. Behavioral parameter estimation relied on indirect inference from aggregate abandonment records; prospective measurement at the individual patient level would substantially strengthen the model’s empirical grounding. Additionally, the current framework addresses single-hospital scheduling and does not account for multi-campus coordination, where patients may choose between facilities based on predicted waiting conditions.

These limitations naturally suggest future research directions. Federated learning architectures could enable multi-campus collaborative scheduling while preserving patient data privacy across institutions. Integration with large language models presents an intriguing opportunity for adaptive patient communication—generating personalized waiting time notifications calibrated to individual behavioral profiles to manage expectations proactively. Most critically, a prospective clinical trial deploying MO-SAC-B alongside existing scheduling systems in a controlled hospital setting would provide the definitive validation that simulation alone cannot offer. We believe such a trial, though logistically demanding, represents the necessary next step toward translating this computational framework into tangible improvements in patient care.

## Data Availability

All data generated and analyzed during the current study are available from the corresponding author upon reasonable request.

## Abbreviations

DRL: Deep Reinforcement Learning
RL: Reinforcement Learning
MDP: Markov Decision Process
SAC: Soft Actor-Critic
PPO: Proximal Policy Optimization
DQN: Deep Q-Network
MO-SAC-B: Multi-Objective SAC with Behavioral Awareness
DES: Discrete-Event Simulation
PER: Prioritized Experience Replay
MWT: Mean Waiting Time
MaxWT: Maximum Waiting Time
UR: Utilization Rate
CSS: Composite Satisfaction Score
NSR: No-Show Rate
FCFS: First-Come-First-Served
WSJF: Weighted Shortest Job First
GA: Genetic Algorithm
